# Zoletil 50-induced mental and behavioral abnormalities: a case report and literature review

**DOI:** 10.3389/fpsyg.2026.1884471

**Published:** 2026-07-08

**Authors:** Yuanji Liu, Shiliang Wang

**Affiliations:** Department of Psychiatry, Huzhou Third Municipal Hospital, The Affiliated Hospital of Wenzhou Medical University, Huzhou, Zhejiang, China

**Keywords:** abuse, addiction, depression, tiletamine e-cigarettes, Zoletil 50

## Abstract

**Background:**

Zoletil 50, mixed with tiletamine and zolazepam, a veterinary anesthetic, has increasingly been used as a substitute drug by substance abusers in recent years. Recently, it has been found in China that tiletamine has been added to electronic cigarettes as a stimulant, highlighting the need for further evaluation of its safety.

**Case presentation:**

A 22-year-old, previously healthy, unmarried male presented to a psychiatric outpatient clinic with a 4-month history of progressive low mood, anhedonia, and feelings of detachment from self and surroundings (depersonalization/derealization). He reported recreational use of powdered Zoletil 50, insufflated approximately 1–2 times per week. Following repeated use, he began experiencing acute, paroxysmal episodes characterized by intense panic, terror, tachycardia, and palpitations, lasting 30–60 min. There was no significant past medical or psychiatric history. Physical examination was unremarkable. Routine blood tests, thyroid function, and ECG were within normal limits.

**Conclusion:**

The use of Zoletil 50 or electronic cigarettes containing tiletamine can lead to addiction, suicidal tendencies, depressed mood, panic attacks. Long-term use of Zoletil 50 may result in various psychiatric and behavioral symptoms, while overdose can be fatal. There is an urgent need for society to strengthen regulations and oversight in this regard.

## 1 Introduction

Zoletil 50 is a veterinary anesthetic supplied as a lyophilized powder in one vial, with a separate solvent for reconstitution; each vial contains 125 mg each of tiletamine and zolazepam (Animal Drugs at FDA, 2026). Its formulation is specifically designed for veterinary use, and it is widely employed for anesthesia in small animals like cats and dogs due to its favorable tolerability and minimal side effects. Tiletamine, an NMDA receptor antagonist structurally similar to ketamine, possesses significant abuse and addiction potential. In recent years, tiletamine has been increasingly used recreationally, often via electronic cigarettes, a trend first documented in 2023 (Zhou et al., 2025).

Potential symptoms of its use include mood alterations, agitation, aggression, suicidal ideation, and dissociative states, while physical examination may reveal muscle tremors, depressed consciousness, increased muscle tone, and hypersalivation. Although originally accessible primarily to veterinary professionals, Zoletil 50 has now spread to individuals with substance use disorders (de la Peña et al., 2014). Nevertheless, documented cases of dependence on this specific agent remain scarce, and this report provides one of the few detailed clinical accounts. This case highlights the urgent need for stringent regulatory control of Zoletil 50 and its active component, tiletamine. Proactive measures are essential to prevent further abuse and mitigate the associated severe psychological and physical harms.

## 2 Case presentation

The patient is a 22-year-old, unmarried male, self-employed. He was admitted to Huzhou Third Municipal Hospital in November 2025 due to “low mood, dissociative experiences, restlessness for 2 months, with recurrent episodes.”

History of present illness: In September 2025, following an argument with a friend, the patient experienced irritability and low mood. He began using tiletamine-containing e-cigarettes. Initial use produced brief euphoria and increased thought speed. However, within 2 days, severe adverse effects emerged: the patient experienced feelings of losing bodily control, somatic dissociation (e.g., “feeling split in half”), limb numbness, a sense of impending doom, palpitations, chest tightness, and marked restlessness. He lost 5 kg within 1 week. This led to his first hospitalization at Hangzhou Seventh People’s Hospital in September 2025, diagnosed with “depressive episode.” Treatment with fluvoxamine and lorazepam resulted in “recovery” at discharge.

After discharge, he intermittently used the same e-cigarette. Later, upon a friend’s introduction, he began directly ingesting Zoletil 50 lyophilized powder, reportedly insufflating approximately 1–2 mg once weekly to chase euphoric effects. Dissociative symptoms and emotional instability persisted. Due to symptom recurrence, he was readmitted to Huzhou Third Municipal Hospital from November 14 to 22, 2025 (second hospitalization), diagnosed with “moderate depressive episode.” Treatment was adjusted (paroxetine, olanzapine, buspirone, lorazepam) with a short course of intravenous diazepam, leading to improvement.

Relapse course: On December 15, 2025, in the context of depressed mood and subjective cognitive decline, the patient intermittently reinitiated oral Zoletil 50 on one or two occasions and also inhaled tiletamine-containing e-cigarette, with a usage frequency of 3–4 times per week, the patient presented to the emergency department with symptoms of excitement, euphoria, loss of bodily control, and suspicion (without persecutory content). He was hospitalized from December 15 to 27, 2025. During this stay, Following three psychological interviews, a relapse prevention plan was formulated, and both the patient and their family were engaged to jointly prevent relapse.

However, although the patient adhered to his medication regimen, his relationship with his parents worsened. He began drinking intermittently, grew increasingly irritable, suffered from insomnia, and withdrew socially, yet he denied significant depressive mood and refused to see his psychiatrist. On January 6, 2026, while alone, he used a small amount of Zoletil 50 again. The following evening (January 6), he experienced an acute, severe panic attack (intense anxiety, fear, impending doom, limb weakness) lasting 30 min. He drove himself to the emergency department and was hospitalized from January 6 to 14, 2026 (fourth hospitalization). The patient reported an inability to control the craving for the substance’s euphoric effects (“chasing the pleasurable experience, feeling unable to control it”).

Investigations: Across hospitalizations, the patient’s height was 182 cm and his body weight fluctuated between 73 kg and 80 kg. Routine investigations—complete blood count, biochemistry, electrocardiogram, chest X-ray, brain MRI, and electroencephalogram—were unremarkable. Urine drug screens for opioids, methamphetamine, and amphetamines were negative. Scores on the Hamilton Depression Rating Scale (HAMD) and Hamilton Anxiety Rating Scale (HAMA) fluctuated during admissions (see Table 1). Vital signs were stable (heart rate 69–85 bpm). Neurological examination revealed no abnormalities detected, tremor, or increased muscle tone.

**Table 1:** HAMD and HAMA scores during hospitalizations.

Date	Diagnose	HAMD	HAMA	Key events	Symptom	Treatment
September 6, 2025	None	None	None	First use of tiletamine-containing e-cigarette	Euphoria, accelerated thought	/
September 2025	Depressive episode	22	14	First hospitalization	Loss of control, separation, numbness, panic, palpitations, tension, depression, weight loss	Fluvoxamine, lorazepam
November 14–22, 2025	Depressive episode	19	None	Second hospitalization 1–2 mg Zoletil 50 lyophilized powder 1–2 times a week and tiletamine-containing e-cigarette	Dissociative symptoms and emotional instability	Paroxetine, olanzapine, buspirone, lorazepam
December 15–27, 2025	Depressive episode and Harmful use of Zoletil 50	18	14	Third hospitalization tiletamine-containing e-cigarette 3 to 4 times per week	Excitement, euphoria, loss of bodily control, suspicion	Paroxetine, quetiapine, lorazepam
January 6–14, 2026	Depressive episode and Harmful use of Zoletil 50	19	16	Fourth hospitalization amount of Zoletil 50	Acute, severe panic attack	Paroxetine, quetiapine, lorazepam

Personal and family history: The patient is an only child with a junior college education and an extroverted personality. He has a 6-year history of nicotine e-cigarette use. He denies other substance abuse or alcohol dependence. Past medical history includes an appendectomy, with no other significant medical conditions. Family history was negative for psychiatric disorders.

## 3 Diagnostic assessment and treatment

Based on the ICD-10 diagnostic criteria, the following diagnoses are considered: Harmful use of Zoletil 50, Moderate depressive episode with somatic symptoms. There are currently no specific treatment guidelines, so management focuses on symptomatic relief. Physical examination ruled out signs such as significant hypersalivation, and the patient denies hallucinations. However, prominent dissociative symptoms alongside a depressed and anxious mood were observed. Therefore, the treatment plan is as follows: Actively improve mood with Paroxetine. Manage medication withdrawal symptoms with agents such as Diazepam and Lorazepam. Provide active education on the dangers of Zoletil 50, informing the patient of its addiction risk.

## 4 Follow-up and outcomes

Following the emergency department visit in January 2026, the patient was discharged with a plan for monthly psychiatric follow-up. During the subsequent 3 months (February to April 2026), the patient attended scheduled monthly outpatient visits. He consistently denied any use of Zoletil 50 or tiletamine-containing e-cigarettes during this period. However, he self-reported irritability, emotional instability, and episodes of transient aggressive behavior directed toward family pets (cats and dogs), without any physical harm to humans. No further depressive mood or suicidal ideation was noted. In response to these symptoms, the outpatient psychiatrist adjusted the medication regimen, transitioning from paroxetine/quetiapine/lorazepam to bupropion (150 mg/day) and extended-release sodium valproate (500 mg/day). Routine liver and kidney function tests remained within normal limits. On May 8, 2026, the patient relapsed after being re-introduced by a friend to a tiletamine-containing e-cigarette. He presented with limb tremors and xanthopsia (yellow-tinted vision). He was subsequently readmitted to the hospital for management of substance-induced symptoms. This fifth admission further illustrates the chronic relapsing nature of tiletamine use disorder, despite periods of abstinence.

## 5 Discussion

Zoletil 50, a veterinary anesthetic documented since 1999, is also subject to human abuse (de la Peña et al., 2014). To systematically review the available literature on human abuse of Zoletil 50 and tiletamine-containing products, we searched PubMed, KoreaMed, Wanfang Data using the terms (“Zoletil” OR “tiletamine” OR “zolazepam”) AND (“misuse” OR “abuse” OR “addiction” OR “case report”). The search yielded 14 relevant articles describing human cases. The drug is most frequently administered via nasal inhalation or intravenous injection. Occasional oral use (Cording et al., 1999), overdose can lead to coma, respiratory depression, and eventually death (de la Peña et al., 2014; Lee et al., 2013). Reported adverse effects include mood instability, irritability, anxiety, suicidal tendencies, aggressive behavior, unresponsiveness, frequent dissociative symptoms, and fragmented visual hallucinations. Common somatic manifestations are tachycardia, tremors of the limbs and jaws, decreased consciousness, hypertension, salivation, and slurred speech, which can also lead to episodic amnesia and ataxia. A review of 14 prior cases revealed that the longest documented duration of use was 6 months, primarily via intravenous injection. Three cases resulted in death, four required ICU admission, one involved a suicidal schizophrenic patient, and in another instance, the drug was administered by the patient’s mother. Twelve cases were for recreational purposes, and nine patients had a confirmed drug abuse history. As human cases are rare, no formal treatment guidelines exist; management is primarily symptomatic, employing agents such as naloxone, diazepam, and lorazepam. Sodium valproate has also been used to stabilize mood and control acute impulsive behavior (detailed in Table 2).

**Table 2:** Cases of human exposure to the TZ combination.

Occupation/Purpose	Clinical presentation/Finding	Sex/Age	Route and dose	Drugs in combination	Duration of Zoletil® use	Country/Year	References
Schizophrenia/suicide	Migraine and abdominal pain; decreased mental status and severe respiratory depression	M/39	Intravenous bolus 1,500 mg (21.4 mg/kg).	None	Not mentioned	Korea/2021	Cha et al. (2021)
Drug abuser/recreational	Dead	M/22	Intravenous bolus 25.1 mg/kg tiletamine and 23.3 mg/kg for zolazepam	Not mentioned	Not mentioned	Korea/2000	Chung et al. (2000)
Drug abuser/recreational	Dead	M/45	Intravenous bolus postmortem blood, urine, and liver concentrations of tiletamine were 295 ng/mL, 682 ng/mL, and 196 ng/g	History of ketamine	Not mentioned	USA/1999	Cording et al. (1999)
Drug abuser/recreational	Vivid auditory hallucination, persecutory delusion, and violent behavior toward his mother.	M/35	Intravenous bolus 15 mg of Zoletil per injection 5–6 times per day at intervals of 4 h	History of ketamine and zolpidem	6 month	China Taiwan/2016	Huang et al. (2016)
Drug abuser/recreational	Involuntary movement of all the 4 limbs	M/35	Not mentioned	History of heroin	2 weeks	China Taiwan/2009	Lee et al. (2009)
Schizophrenic/daughter injected her	Stupor, hypotension, bradycardia and dead	F/83	Intravenous bolus seven vials of “Zoletil 50”	None	None	Korea/2013	Lee et al. (2013)
Drug abuser/recreational	a brief period of tonic–clonic movements and unconsciousness	M/60	Intravenous bolus	History of cocaine	None	Australia/2024	Page et al. (2024)
Drug abuser/recreational	Aggressive behavior, irritability, drooling, transient amnesia	M/48	Intravenous bolus	History of cocaine	none	Australia/2024	Page et al. (2024)
Veterinary worker/recreational	Obtunded and hypotensive but had an intact gag reflex	F/30	Intravenous bolus 200 mg of telazol, or 100 mg of each ingredient	None	Not mentioned	USA/2001	Quail et al. (2001)
Work at a veterinarian’s office/recreational	Slurred speech, injected sclera, nystagmus on lateral gaze, and 3 mm reactive pupils. Within an hour of presentation, the patient became combative and violent requiring restraints and sedation	M/16	Drink 500 mg of Telazol®	None	None	USA/2002	Redmond and Herman (2002)
Drug abuser/recreational	Abundant visions, dissociation, ataxia, muscle jerking, inability to walk and lung infection, along with changes of consciousness.	M/33	Smoking once a week.	History of methamphetamine and gamma-hydroxybutyrate	4 months	China/2023	Song et al. (2023)
History of insomnia for 1 year/recreational	Involuntary tremor of the lower jaw, lethargy, failing eyesight dribbling saliva feeling chill and fatigue	M/30	Intravenous bolus self-administered IV injection of 9 ampoules of Terazol (2,250 mg) within 9 days	None	2 weeks	Korea/2013	Wang et al. (2013)
Chef, history of attention deficit hyperactive disorder, oppositional defiance disorder /recreational	Acute delirium unresponsive increased limb muscle tone high blood pressure	M/21	Nasal inhalation positive in urine test	History of ketamine	Not mentioned	Hong Kong (China)/2020	Yip et al. (2020)
Drug abuser/recreational	Dissociative symptoms, visual hallucinations, depressive moods, suicidal thoughts and attempts, poor appetite, sleep disturbance, unsteady gait, limb tremors, head tremors, voice tremors, dysarthria, irritability, anxiety, chest tightness, nausea, dizziness	M/40	Tiletamine-containing e-cigarettes increased from several times a week to daily within a month, with each session escalating from two to eight cartridges per day, with the exact dosage per cartridge unknown	History of ketamine and heroin and heroin and alcohol use and etomidate	5 month	China/2025	Zhou et al. (2025)

To the best of our knowledge, this is a rare case involving tiletamine-containing e-cigarette and Zoletil 50 use in a patient with no prior substance dependence (aside from smoking) and no pre-existing psychiatric diagnosis, distinguishing it from previously reported cases that involved high-dose use and a history of other substance use disorders (e.g., methamphetamine, ketamine) (Zhou et al., 2025; Page et al., 2024; Song et al., 2023). The patient exhibited poor emotional stability, depression, aggressive tendencies, palpitations, and dissociation-like symptoms, but did not show significant muscle tremors or withdrawal symptoms. Urine examination was performed, which showed that amphetamine, morphine, methamphetamine, etc. were negative. Since the patient has been using lorazepam intermittently outside the hospital, blood tests for benzodiazepines were not performed. Given the patient’s lack of relevant medical history and the absence of obvious withdrawal symptoms during several hospitalizations, it was considered that the patient did not have obvious abuse of other substances. The patient was hospitalized four times and recovered and was discharged from the hospital. However, the patient repeatedly took Zoletil 50, so it was considered that the patient had developed dependence on Zoletil 50.

Zoletil 50 (Telazol) is a commonly used veterinary anesthetic (Kim et al., 2016). Its active ingredients are tiletamine and zolazepam. Tiletamine exhibits structural similarity to ketamine (Walzer and Huber, 2002), tiletamine exhibits structural similarity to ketamine, and prior animal studies have demonstrated ketamine-like conditioned place preference (CPP) and self-administration (SA) behaviors, Zolazepam has also been shown to produce clear CPP and SA in animals following repeated administration, and pre-exposure to the drug can influence addictive behaviors (Noda et al., 1998; Noda et al., 2004). This may explain why patients with a history of ketamine-class drug use are more likely to use Telazol 50 (Walzer and Huber, 2002). Side effects observed in animal experiments include tachycardia, involuntary muscle twitches, salivation, and transient hypothermia. Dose-dependent respiratory depression, instability, and prolonged resuscitation time were also reported. Additional effects included (Lefkov and Müssig, 2007) myocardial depression and hypothermia persisting for several days, as well as seizures (Page et al., 2024; Hunt et al., 2020). It is classified as a controlled substance in the United States and South Korea due to its harmful societal effects (Santo et al., 2022).

Based on previous reports, the use of Zoletil 50 has primarily been associated with recreational purposes, often involving regular or high-dose administration. These accounts have documented symptoms such as emotional instability, tremors, and dissociative-like experiences. At high doses, respiratory depression, pneumonia, altered states of consciousness, and fatalities have been observed. In the present case, the patient exhibited marked low mood, panic-like episodes, and dissociative experiences. Given that each depressive episode and hospitalization occurred following the inhalation of Zoletil 50 or tiletamine-containing e-cigarettes, a potential drug-related etiology is considered. The following factors are hypothesized: (1) The patient experienced transient suicidal ideation after smoking e-cigarettes 3–4 times per week. (2) Previous studies have reported a higher prevalence of affective disorders among individuals with substance dependence (Hunt et al., 2020), suggesting a degree of shared underlying mechanisms (Santo et al., 2022). (3) The use of Zoletil 50 has been associated with emotional instability, dissociative experiences, psychotic symptoms, euphoria, and aggressive impulses, all of which were observed in this case. Therefore, it is plausible that Telazol-50 may contribute to affective abnormalities.

Currently, there is no established treatment for the abuse of Zoletil-50. Previous case reports have suggested the use of medications such as mecobalamin, and primidone for tremor control; symptomatic management of mood changes; valproate sodium for agitation control; and lorazepam as substitution therapy (Song et al., 2023; Quail et al., 2001). In clinical practice, antidepressant use during depressive episodes is not unusual among patients with substance use disorders (Hunt et al., 2020). In this case, paroxetine was administered to improve the patient’s mood, while diazepam was used short-term to address panic and suicidal ideation, with a gradual transition to lorazepam. Despite three in-hospital psychological interview sessions, the patient was attended monthly follow-ups irregularly after discharge and relapsed. Thus, the non-pharmacological component of this case was unfortunately unsuccessful. This reflects the difficulty of translating brief inpatient interventions into long-term abstinence, particularly in patients with poor motivation and insufficient family support. This limitation underscores the need for more robust post-discharge follow-up and structured psychosocial rehabilitation.

In summary, this case report presents a 22-year-old male, non-veterinary worker, with no history of alcohol or tobacco dependence, who developed psychiatric and behavioral symptoms after initially using electronic cigarettes containing tiletamine for 4 months, followed by the use of Zoletil 50. This case highlights that in patients presenting with acute psychiatric and behavioral changes, the possibility of substance use should be considered even in the absence of a significant prior history of substance dependence. However, this case report also has certain limitations. The substance dosage was based on patient self-report without objective verification. Non-pharmacological therapies (e.g., CBT, motivational interviewing) were not fully delivered, and the patient was lost to follow-up after discharge. Longer follow-up is needed to assess long-term outcomes and relapse prevention.

## Data Availability

The original contributions presented in the study are included in the article/supplementary material, further inquiries can be directed to the corresponding author.
